# Early-life stress induces persistent alterations in 5-HT_1A_ receptor and serotonin transporter mRNA expression in the adult rat brain

**DOI:** 10.3389/fnmol.2014.00024

**Published:** 2014-04-10

**Authors:** Javier A. Bravo, Timothy G. Dinan, John F. Cryan

**Affiliations:** ^1^Grupo de NeuroGastroBioquímica, Laboratorio de Química Biológica, Instituto de Química, Facultad de Ciencias, Pontificia Universidad Católica de ValparaísoValparaíso, Chile; ^2^Department of Psychiatry, University College CorkCork, Ireland; ^3^Laboratory of Neurogastroenterology, Alimentary Pharmabiotic Centre, University College CorkCork, Ireland; ^4^Department of Anatomy, University College CorkCork, Ireland

**Keywords:** maternal separation, 5-HT_1A_ receptor, serotonin transporter, amygdala, dorsal raphé nucleus

## Abstract

Early-life experience plays a major role in the stress response throughout life. Neonatal maternal separation (MS) is an animal model of depression with an altered serotonergic response. We hypothesize that this alteration may be caused by differences in 5-HT_1A_ receptor and serotonin transporter (SERT) mRNA expression in brain areas involved in the control of emotions, memory, and fear as well as in regions controlling the central serotonergic tone. To test this, Sprague–Dawley rats were subjected to MS for 3 h daily during postnatal days 2–12. As control, age matched rats were non-separated (NS) from their dams. When animals reached adulthood (11–13 weeks) brain was extracted and mRNA expression of 5-HT_1A_ receptor in amygdala, hippocampus and dorsal raphé nucleus (DRN) and SERT in the DRN was analyzed through *in situ* hybridisation. Densitometric analysis revealed that MS increased 5-HT_1A_ receptor mRNA expression in the amygdala, and reduced its expression in the DRN, but no changes were observed in the hippocampus in comparison to NS controls. Also, MS reduced SERT mRNA expression in the DRN when compared to NS rats. These results suggest that early-life stress induces persistent changes in 5-HT_1A_ receptor and SERT mRNA expression in key brain regions involved in the development of stress-related psychiatric disorders. The reduction in SERT mRNA indicates an alteration that is in line with clinical findings such as polymorphic variants in individuals with higher risk of depression. These data may help to understand how early-life stress contributes to the development of mood disorders in adulthood.

## INTRODUCTION

In the early postnatal period of the rat, the brain is thought to be a developmental equivalent to the last trimester *in utero* and the perinatal period of human brain development (Romijn et al., 1991; Watson et al., 2006; Goodfellow et al., 2009), thus allowing for the use of postnatal rodent models in the investigation of the early programing of stress-related psychiatric disorders. It has been proposed that stress during developmental stages, can lead to developmental alterations that become evident in adult life (Barker, 1995), and moreover, during early-life the psychosocial milieu can substantially alter the nervous system, through mechanisms that permanently affect gene expression (Mathews and Janusek, 2011).

In the central nervous system (CNS), one of the key neurotransmitter systems involved in the response to stress and in the development of neuropsychiatric disorders is the 5-hydroxytriptamine (5-HT) system (Kirby et al., 1995; Graeff et al., 1996; Cryan et al., 2005; Savitz et al., 2009; O’Leary and Cryan, 2010). The majority of 5-HT neurons are located in the dorsal and median raphé nucleus in the brainstem (Graeff et al., 1996; Michelsen et al., 2007). Projections from these neurons innervate several structures of the limbic system, including the amygdala and hippocampus, and it has been described that through these projections the 5-HT system regulates the fight or flight reaction to stress (Graeff et al., 1996; Michelsen et al., 2007), by a region-specific release of 5-HT (Kreiss and Lucki, 1994; Kirby et al., 1995; Graeff et al., 1996).

There are 14 types of 5-HT receptors, divided into seven families, with different subtypes identified by letters (A–F in the case of 5-HT_1_ receptors; Hoyer et al., 1994; Barnes and Sharp, 1999; Bockaert et al., 2010). One of these receptors is 5-HT_1A_, a G protein-coupled receptor that has been described to play an important role in the development of psychiatric disorders (Bowen et al., 1989; López et al., 1998; Drevets et al., 1999; Gross et al., 2002; Savitz et al., 2009). The 5-HT_1A_ receptor is predominantly a somatodendritic autoreceptor in the neurons of the raphé nucleus regulating the amount of 5-HT released and therefore serotonergic activity in the different projection areas (Blier and de Montigny, 1987; Hutson et al., 1989; Hjorth and Sharp, 1991; Kreiss and Lucki, 1994; Savitz et al., 2009). Also, 5-HT_1A_ receptor expression has been described in forebrain areas (Chalmers and Watson, 1991; Pompeiano et al., 1992; Cryan et al., 2005; Savitz et al., 2009) including the hippocampus and amygdala, structures involved in learning, control of emotions, memory and fear related information (Vizi and Kiss, 1998; Nestler et al., 2002; LeDoux, 2007). Alterations in 5-HT_1A_ receptor function have been related to mood disorders, as imaging analysis shows that depressive patients have reduced 5-HT_1A_ receptor binding (Drevets et al., 1999; Sargent et al., 2000) as well as blunted responses to 5-HT_1A_ receptor agonists (Lesch et al., 1990a,b).

Another component of the 5-HT system is the serotonin transporter (SERT), a presynaptic protein involved in the termination of the serotonergic signal through the reuptake of 5-HT from the synapse (Blakely et al., 2005). In the pharmacological treatment of depression, selective serotonin reuptake inhibitors (SSRIs) have been widely used (Frazer, 1997). SSRIs can readily inhibit SERT activity and elevate the serotonergic tone in the brain. However, full therapeutic effects become apparent only after chronic SSRI use, suggesting that alterations in this transporter are highly relevant to the development and treatment of psychiatric disorders (Frazer and Benmansour, 2002).

Neonatal maternal separation (MS) is a well validated animal model of depression and increases anxiety resulting in behavioral alterations (Lippmann et al., 2007) and functional changes in the hypothalamus-pituitary-adrenal (HPA) axis responsiveness in adulthood (Ladd et al., 1996; Schmidt et al., 2004; O’Mahony et al., 2009). In addition, MS animals have been reported to display alterations in their central corticotrophin releasing factor (CRF) system (Bravo et al., 2010; O’Malley et al., 2011), which is suggestive of an altered gene expression in key brain areas as result of early-life stress.

There is evidence suggesting an enhanced serotonergic response in animals subjected to MS, as there are differences in brain stem levels of 5-HT and its metabolite 5-hydroxyindole acetic acid (5-HIAA; O’Mahony et al., 2008), as well as increased responsiveness to the SSRI citalopram (Arborelius et al., 2004).Therefore, differences in central serotonergic modulation in adult rats subjected to early-life stress could arise as a result of altered 5-HT_1A_ receptor and SERT expression in areas of the brain involved in the control of emotions, memory, and fear as well as in areas controlling the central serotonergic tone. To test this, *in situ* hybridization was used to study topographical differences in 5-HT_1A_ receptor and SERT mRNA expression in the hippocampus, amygdala, and dorsal raphé nucleus (DRN) between MS and non-separated (NS) rats.

## MATERIALS AND METHODS

### ANIMALS

Adult male Sprague–Dawley (SD) rats that underwent a MS protocol were used (*n* = 6 MS from three different litters and *n* = 6 NS controls from three different litters). All animals were housed in standard conditions (room temperature of 21°C, with a 12 h light dark cycle) with access to regular chow and water *ad libitum*. Cages were cleaned once weekly to avoid excessive handling. Rats were of comparable weight (276–410 *g*) and age (11–13 weeks) at the moment of sacrifice All experimental procedures were carried out in accordance with the protocols approved by the Ethics Committee, at University College Cork, Cork, Ireland under a license issued from the Department of Health and Children (Cruelty to Animal Act 1876, Directive for the Protection of Vertebrate Animals used for Experimental and other Scientific Purposes [89/609/EEC]).

### MATERNAL SEPARATION

Early-life stress procedure (Hyland et al., 2009; O’Mahony et al., 2009, 2010) was adapted from a previously described protocol (Wigger and Neumann, 1999). Briefly, the litters that were randomly assigned to undergo MS, were removed from the home cage and placed into a smaller cage on heating pads set at 30–33°C for 3 h (9.00–12.00 h). After that time, pups were returned to the original home cage in the main colony room. This procedure was repeated from postnatal day 2 (P2) to P12. Control, NS litters remained undisturbed except for routine cage cleaning performed once a week. At P21, pups were weaned and group-housed (3–5 per cage), and left undisturbed until adulthood (11–13 weeks). We have previously shown that this MS protocol induces an array of behavioral and physiological changes that are indicative of increased anxiety and altered HPA axis function (O’Mahony et al., 2009).

### SACRIFICE AND *IN SITU* HYBRIDISATION

Animals were lightly anesthetized with isoflurane, and killed by decapitation. The brain was immediately extracted and snap frozen in isopentane kept cold with dry ice. The brains were stored at -80°C before being processed for *in situ* hybridisation.

The *in situ* hybridisation was carried out with oligodeoxynucleotide (cDNA) probes complementary to 5-HT_1A_ receptor mRNA (2107–2151 pb access number AF217200)and SERT mRNA (1719–1763 pb access number Y11024.1), labeled with a digoxigenin (DIG) oligonucleotide 3′-OH tailing kit (Roche, Molecular Biochemicals, Mannheim, Germany). The hybridisation was conducted as previously described (Bravo et al., 2009, 2010). Briefly, coronal brain sections of 10 μm thick were obtained from frozen brains and mounted on superfrost-plus glass slides (Menzel-Glaser, Menxel GmbH & Co., Germany). For hippocampus, four to five non-consecutive slices separated at least 100 μm from each other, approximately from bregma -2.56 mm to bregma -3.6 mm were analyzed bilaterally. For the amygdala: bilateral analysis of four to five slices of tissue, separated at least 100 μm from each other, approximately from bregma -1.80 mm to bregma -2.80 mm. In the case of the DRN at least three slices separated as a minimum as 100 μm from each other, approximately from bregma -7.64 mm to bregma -8.00 mm were obtained. These sections were post-fixed in 4% paraformaldehyde made in PBS for 30 min. Then the slides were permeabilized with proteinase K (0.5 mg/100 mL in TE buffer) and treated with acetic anhydride buffer. Next, the slides underwent dehydration through a series of ethanol dilutions (70, 95, and 100%) before being delipidated in chloroform for 5 min. The tissues were then rehydrated and placed in a humidity chamber with the hybridisation solution [formamide 50%, saline sodium citrate (SSC) buffer 4x, sheared salmon DNA 6.25 mg/mL, tRNA 125 μg/mL, and cDNA probe at fixed concentration of 100 pmol/mL for each probe] and incubated overnight at 37°C. After that, the sections were washed in ascending dilutions of SSC buffer (4, 2, 1, and 0.5x), and then equilibrated with maleic acid 0.1 M buffer before blocking for unspecific protein binding with Roche’s blocking reagent (Roche, Molecular Biochemicals, Mannheim, Germany). After 30 min of blocking, the DIG molecules attached to the hybridized probes were detected with an anti DIG antibody, conjugated with an alkaline phosphatase (Roche, Molecular Biochemicals, Mannheim, Germany). Finally, a substrate for the alkaline phosphatase (NBT/BCIP; Sigma, St. Louis, MO, USA) was added, and when a violet/blue precipitate was present on the tissues, the reaction was stopped. The slides were then left to air dry and cover-slips were mounted with DPX mounting media (Fisher Scientific, Loughborough, UK). Once the mounting media was dry, pictures of the areas of interest were taken with an Olympus DP71 digital camera attached to an Olympus BX51 microscope (Olympus Corporation, Tokyo, Japan). Specificity of the hybridisation was evaluated by the use of 100-fold excess of the unlabelled oligodeoxynucleotide. For semiquantitative analysis, densitometric measurements of each hippocampal, amygdala, and DRN were analyzed using FujiFilm’s Science Lab Multi Gauge v2.2 software (Fuji Photo Film Co., Ltd). All pictures were analyzed in gray scale and the value given by the software corresponds to the intensity of pixels (the darkest staining is the highest intensity; and the lightest staining the lowest intensity) in a given area (density of pixels). In the hippocampus, the hybridisation signal in the *stratum radiatum* was considered as background and was subtracted from the pixel density values obtained in the hippocampal cell layers. As for the amygdala, a small region between the analyzed areas was considered as background, and for the DRN a small region surrounding this structure was taken as background. For each animal the value represents the average from 4–5 non-consecutive brain sections (analyzed on both brain hemispheres for hippocampus and amygdala).

### STATISTICAL ANALYSIS

All the values are expressed as the mean ± SEM. Data were analyzed with a two tailed Student’s *t*-test using GraphPad Prism 4 (GraphPad Software Inc., La Jolla, CA, USA). Statistical significance was accepted at the level *p* < 0.05.

## RESULTS

**FIGURE 1:**
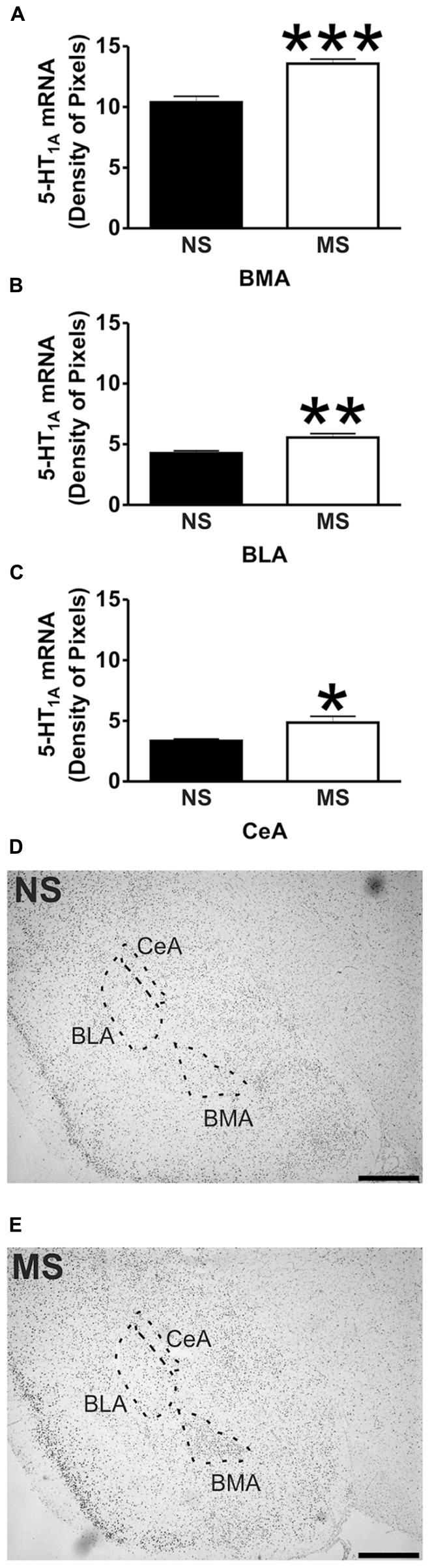
**5-HT_1A_ receptor mRNA expression in the amygdala.** Maternal separation (MS) increases 5-HT_1A_ receptor mRNA expression in three different areas of the amygdala in comparison to non-separated (NS) animals. Graphical representations of the densitometric analysis in the basomedial amygdala (BMA; ***p < 0.001; **A**), basolateral amygdala (BLA; **p < 0.01; **B**), and central amygdala (CeA; *p < 0.05; **C**). Representative microphotographs of 5-HT_1A_ receptor mRNA expression in NS **(D)** and MS **(E)** animals (scale bar represents 1 mm; NS, *n* = 6 and MS, *n* = 6).

**FIGURE 2:**
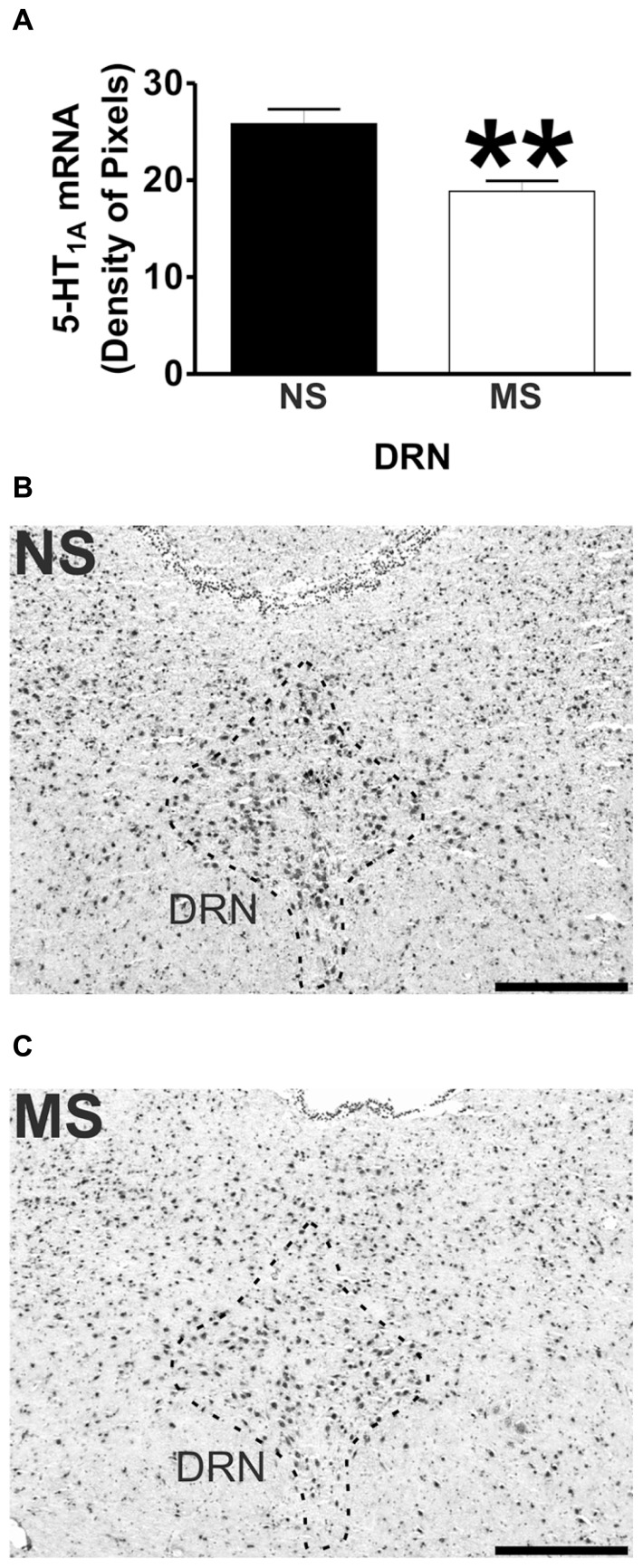
**5-HT_1A_ receptor mRNA expression in the DRN.** Maternal separation (MS) reduces 5-HT_1A_ receptor mRNA expression in the DRN in comparison to NS animals. Graphical representations of the densitometric analysis in the DRN (**p<0.01; **A**). Representative microphotographs of 5-HT_1A_ receptor mRNA expression in NS **(B)** and MS **(C)** animals (scale bar represents 500 μm; NS, *n* = 6 and MS, *n* = 6).

**FIGURE 3:**
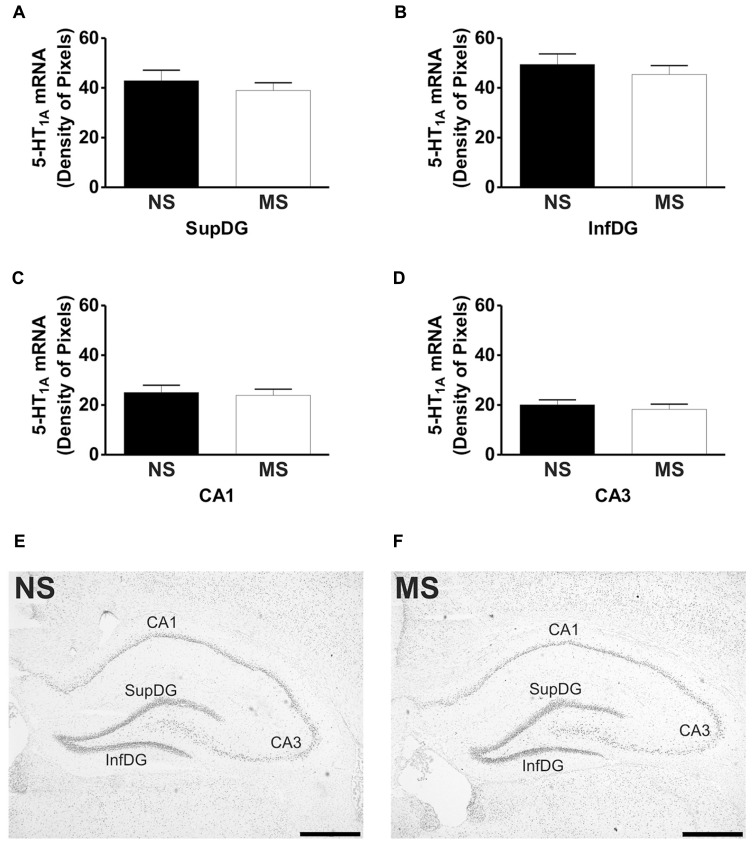
**Hippocampal expression of 5-HT_1A_ receptor mRNA.** Maternal separation (MS) did not affect 5-HT_1A_ receptor mRNA expression in the hippocampus when compared to NS animals. Graphical representations of the densitometric analysis in the suprapyramidal layer of the dentate gyrus (SupDG; **A**), infrapyramidal layer of the dentate gyrus (InfDG; **B**), cornus ammon field 1 (CA1; **C**) and cornus ammon field 3 (CA3; **D**). Representative microphotographs of 5-HT_1A_ receptor mRNA expression in NS **(E)** and MS **(F)** animals (scale bar represents 1 mm; NS, *n* = 6 and MS, *n* = 6).

**FIGURE 4:**
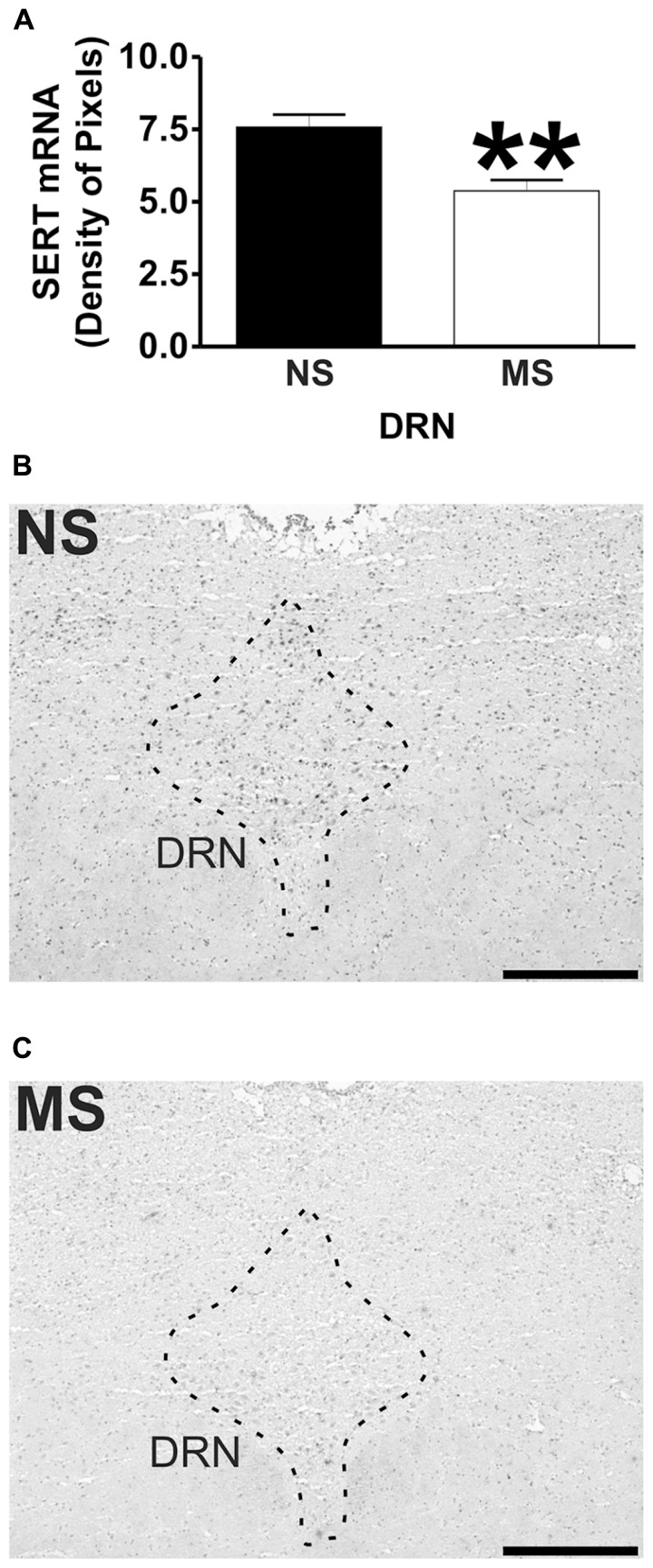
**SERT mRNA expression in the DRN.** Maternal separation (MS) reduces SERT mRNA expression in the DRN in comparison to NS animals. Graphical representations of the densitometric analysis in the DRN (**p < 0.01; **A**). Representative microphotographs of SERT mRNA expression in NS **(B)** and MS **(C)** animals (scale bar represents 500 μm; NS, *n* = 6 and MS, *n* = 6).

Signal for 5-HT_1A_ receptor mRNA was detected in the amygdala (**Figures 1D,E**), DRN (**Figures 2B,C**), and hippocampus (**Figures 3E,F**), and for SERT mRNA in the DRN (**Figures 4B,C**). The level of staining in each case allowed densitometric analysis. Negative controls were performed using an excess of unlabelled cDNA probe during the hybridisation stage (not shown).

### 5-HT_1A_ RECEPTOR mRNA EXPRESSION

Early-life stress significantly increased the levels of 5-HT_1A_ receptor mRNA in the basomedial amygdala (BMA; **Figure 1A**; NS vs. MS: 10.39 ± 0.49 vs. 13.58 ± 0.36; *t*(10) = 5.226, *p* < 0.001), basolateral amygdala (BLA; **Figure 1B**; NS vs. MS: 4.27 ± 0.18 vs. 5.55 ± 0.33; *t*(10) = 3.373, *p* < 0.01) and central amygdala (CeA; **Figure 1C**; NS vs. MS: 3.35 ± 0.14 vs. 4.86 ± 0.51; *t*(10) = 2.838, *p* < 0.05), and decreased the expression of this transcript in the DRN (**Figure 2A**; NS vs. MS: 25.85 ± 1.49 vs. 18.87 ± 1.1; *t*(10) = 3.804, *p* < 0.01) when compared to NS controls. However, densitometric analysis of the 5-HT_1A_ receptor transcript revealed no differences between MS and NS animals in any of the hippocampal layers (**Figure 3**).

### SERT mRNA EXPRESSION

Maternal separation induced a significant reduction to the transcript for SERT in the DRN in comparison to NS rats (**Figure 4A**; NS vs. MS: 7.58 ± 0.43 vs. 5.38 ± 0.37; *t*(10) = 3.840, *p* < 0.01).

## DISCUSSION

The present data shows that early-life stress affects gene expression in adulthood, contributing to inadequate stress responses and could therefore lead to the manifestation of stress-related psychiatric disorders. Similar alterations have been described for another neurotransmitter system (Bravo et al., 2010), which further suggest that early-life stress does affects CNS function. Although MS did not affect 5-HT_1A_ receptor mRNA expression in the hippocampus, a structure involved in memory and learning (Jacobson and Sapolsky, 1991; Vizi and Kiss, 1998), it increased its expression in three subregions of the amygdala, a structure related to the control of emotions and fear (LeDoux, 2007). In addition, MS reduced 5-HT_1A_ receptor mRNA expression in the DRN, the major source of serotonergic input to the forebrain which is involved in the control of the central serotonergic tone (Graeff et al., 1996). Also, early-life stress reduced the expression of SERT mRNA in the DRN, which could have an impact on the bioavailability of 5-HT in projection areas of the DRN. These alterations suggest that the behavioral, physiological and molecular deficits described for this animal model (Ladd et al., 1996; Schmidt et al., 2004; Lippmann et al., 2007; O’Mahony et al., 2009) could arise as a consequence of changes in gene expression in key brain regions involved in the development of stress-related psychiatric disorders.

Early-life stress, such as that induced by MS, physical, sexual and emotional abuse and general neglect during childhood, has been associated with serious psychiatric impairments in adulthood (MacMillan et al., 2001; Lupien et al., 2009). During postnatal development the brain undergoes a variety of adaptive changes that depend mostly on the type of stimuli being received (Schmidt et al., 2004). In rats there is a period of reduced stress responsiveness during the first 2 weeks of life (Sapolsky and Meaney, 1986) which can be disinhibited by prolonged MS (Schmidt et al., 2004; O’Mahony et al., 2009). These long periods of MS immediately impact brain gene expression, including the 5-HT_1A_ receptor. For example, Goodfellow et al. (2009) show that 5-HT_1A_ electrophysiological activity in the prefrontal cortex is enhanced in the first 2 to 3 postnatal weeks after MS (3 h a day from P2 to P14). Moreover, mRNA expression of the 5-HT_1A_ receptor in maternally separated animals is increased at postnatal day 9 (Goodfellow et al., 2009). However, when these animals reach adulthood (≥P40), the electrophysiological effects mediated by the 5-HT_1A_ receptor are not different between maternally separated rats and their respective controls, and there is no difference in mRNA expression between rats subjected to early-life stress and control animals (Goodfellow et al., 2009). Nevertheless, exposure to social isolation stress reduces the 5-HT_1A_-elicited electrophysiological activity in the prefrontal cortex of animals that were exposed to early-life stress, in comparison to control rats (Goodfellow et al., 2009), thus suggesting that early-life stress increases the susceptibility toward stress-related psychiatric disorders in adulthood.

We have previously described alterations in serotonin metabolism in MS rats (O’Mahony et al., 2008), and additionally, alterations in the central serotonergic system have also been described as a result of different MS procedures (Arborelius and Eklund, 2007; Oreland et al., 2009). Oreland et al. (2009) have shown that brief exposures to MS (15 min from P1 to P13) reduces brain stem expression of 5-HT_1A_ receptor mRNA (Oreland et al., 2009), a procedure that can also affect other neurotransmitter systems (Jaworski et al., 2005; Plotsky et al., 2005). This type of MS could be considered a more naturalistic stress as it mimics the natural rearing environment of rats, where the mother leaves her pups for short periods to forage (Arborelius et al., 2004; Arborelius and Eklund, 2007; Oreland et al., 2009). On the other hand, there is also evidence demonstrating that brief and long daily periods of MS do not affect 5-HT_1A_ receptor and SERT mRNA expression (Arborelius et al., 2004), and furthermore, it has been shown that long periods of daily MS are more effective in producing changes in behavior and alterations in biomarkers associated to stress-related psychiatric disorders (Lippmann et al., 2007; O’Mahony et al., 2009; Bravo et al., 2010). Our previous studies demonstrated that a protocol consisting of 3 h of MS from P2 to P12 produces an increase in corticosterone levels (O’Mahony et al., 2009) and an increase in serotonin turnover (O’Mahony et al., 2008). However, quantitative real time PCR (qRT-PCR) revealed no differences in the expression of 5-HT_1A_ receptor and SERT transcripts in complete brainstem homogenates of MS animals in comparison to NS rats (O’Mahony et al., 2008). Whilst qRT-PCR is a sensitive technique used to assess gene expression, is also a crude method which dilutes any localized changes in gene expression that might occur as a result of early-life stress. Therefore, the present findings corroborate that alterations in serotonin metabolism, induced by MS (O’Mahony et al., 2008), can be consequence of changes in gene expression within the DRN. However, it is important to note that the present observations only represent changes at the mRNA level and not protein, and they could be just a reflection of a more complex situation involving other neurotransmitter systems (Bravo et al., 2010) and a variety of intracellular cascades that can affect the expression of these transcripts in the different studied areas.

Maternal separation affected the expression of 5-HT_1A_ receptor mRNA in the amygdala. The transcript for this receptor has been described in the rat BMA (Chalmers and Watson, 1991; Pompeiano et al., 1992), and binding of radio labeled 5-HT_1A_ receptor antagonists has been shown in the BLA and CeA (Vicentic et al., 2006). In the present study, early-life stress increased the levels of the transcript for 5-HT_1A_ receptor in the BMA, and also in the BLA and CeA, although in these areas the level of transcript was much lower than in the BMA. It has been shown that activation of 5-HT_1A_ receptors within the amygdala using the agonist 8–Hydroxy-2-(dipropylamino)tetralin (8-OH-DPAT) reduces the levels of social interaction of male rats (Gonzalez et al., 1996), demonstrating that 5-HT_1A_ activation in the amygdala of rats mediates anxiogenic effects. Moreover, and in line with the present findings, Vicentic et al. (2006) showed that non-handled rats (similar to our NS condition) have lower binding capacity of the 5-HT_1A_ receptor antagonist 4-(2′-methoxyphenyl)-1-[2′-[*N*-(2′′-pyridinyl)-*p*-iodobenzamido] ethylpiperazine (pMPPI) in the BMA and BLA in comparison to MS animals. Therefore, the increase in 5-HT_1A_ receptor mRNA expression within the amygdala of MS rats could account for some of the behavioral changes observed by Lippmann et al. (2007), where MS reduced locomotor activity, increased acoustic startle and also affects HPA axis responsiveness in adulthood (Ladd et al., 2005; Lippmann et al., 2007; O’Mahony et al., 2009).

In contrast to the increased expression of 5-HT_1A_ receptor mRNA in the amygdala, there were lower levels of 5-HT_1A_ receptor mRNA found in the DRN of MS rats in comparison to their controls. In this structure there is a high density of 5-HT_1A_ receptors (Blier and de Montigny, 1987; Hutson et al., 1989; Hjorth and Sharp, 1991; Kreiss and Lucki, 1994; Cryan et al., 2005; Savitz et al., 2009), and activation of these presynaptically located receptors decreases the firing frequency, 5-HT synthesis and release from these neurons (Blier and de Montigny, 1987; Sprouse and Aghajanian, 1987; Hjorth and Magnusson, 1988; Hutson et al., 1989; Sharp et al., 1989; Kreiss and Lucki, 1994; Cryan et al., 2005). In addition, 5-HT_1A_ receptor activation in the DRN has been shown to produce anxiolytic effects in different animal models (Andrews et al., 1994; Hogg and File, 1994; Jolas et al., 1995; Picazo et al., 1995; File et al., 1996; Remy et al., 1996; Romaniuk et al., 2001; Koprowska et al., 2002). The reduced levels of 5-HT_1A_ receptor mRNA in the DRN of MS rats suggests an impaired regulation of the central serotonergic tone that could translate into inadequate behaviors toward stressful situations such as those observed by Lippmann et al. (2007). Moreover, we have previously shown that MS rats have altered 5-HT and 5-HIAA levels in the brain stem that clearly suggests an increased turnover of this neurotransmitter (O’Mahony et al., 2008). This could be a consequence of the lower 5-HT_1A_ receptor expression in the DRN, as a lower level of this receptor could impact on the frequency of discharge and/or synthesis and release of 5-HT and therefore affect the neurotransmitter’s metabolism. In line with the previous suggestion, Ase et al. (2000) showed that 5-HT_1A_ receptor knock-out mice have increased 5-HT turnover. However, these animals show no differences in basal levels of 5-HT in forebrain areas (Ase et al., 2000; He et al., 2001; Knobelman et al., 2001) and in the DRN (Ase et al., 2000; Bortolozzi et al., 2004) in comparison to wild-type controls. These observations argue against a role of presynaptic 5-HT_1A_ receptors in the maintenance of the central serotonergic tone, and therefore reveal the complexity of 5-HT neurotransmission regulation.

Another level of regulation to the central 5-HT neurotransmission involves SERT. The levels of SERT mRNA were lower in the DRN of MS rats in comparison to NS rats, which suggest that the reuptake of the neurotransmitter could be affected. The importance of this finding is that changes in SERT expression have been related to psychiatric disorders. For instance, during treatment with SSRIs, the most widely prescribed antidepressants (Frazer, 1997), SERT gets downregulated, which seems to correlate with the efficacy of the treatment (Benmansour et al., 1999, 2002; Frazer and Benmansour, 2002; Gould et al., 2003; Thakker et al., 2004, 2005). However, SERT deficient mice display anxiety- and depression-like behaviors (Holmes et al., 2002; Lira et al., 2003), which suggest that the absence of this gene from early developmental stages affects the ability to cope with stressful situations throughout life. In addition, animals treated during early development with SSRIs also display altered behaviors in adulthood (Mirmiran et al., 1981; Vogel et al., 1990), as the antidepressant would down regulate SERT in early-life. Moreover, downregulation of SERT in adult animals resembles the effects of antidepressant treatment (Thakker et al., 2004, 2005), further highlighting an important role of SERT in the development of the serotonergic system during early-life. In the present study, the reduction in SERT mRNA expression in the DRN not only could affect local serotonin levels (and perhaps its turnover), but it could also impact the adequate development of the serotonergic system and therefore affect the ability to cope with stress. In addition, the reduction in SERT mRNA indicates an alteration that is in line with clinical findings. Individuals with a short allele for SERT, that reduce the efficiency of the gene’s transcription, showed more depressive symptoms in relation to stressful events than individuals with the long version of the allele (Caspi et al., 2003), and therefore are at a higher risk of developing psychiatric disorders such as depression.

In summary, the present findings, along with previous observations on other neuronal systems (Bravo et al., 2010) strongly suggest that early-life stress permanently affects gene expression in the CNS. These changes in 5-HT_1A_ receptor and SERT mRNA reflect alterations in a neurotransmitter system that has been extensively related to the development of mood disorders. Moreover, these changes were observed in key brain areas related to the behavioral response to stress. Therefore, these data helps to understand how early-life stress contributes to the development of mood disorders later in life.

## AUTHOR CONTRIBUTIONS

Javier A. Bravo, Timothy G. Dinan and John F. Cryan designed research; Javier A. Bravo performed research and acquired data; Javier A. Bravo, Timothy G. Dinan and John F. Cryan interpreted and analyzed data; and Javier A. Bravo, Timothy G. Dinan and John F. Cryan drafted, revised and wrote the paper.

## Conflict of Interest Statement

The authors declare that the research was conducted in the absence of any commercial or financial relationships that could be construed as a potential conflict of interest.
